# "Food company sponsors are kind, generous and cool": (Mis)conceptions of junior sports players

**DOI:** 10.1186/1479-5868-8-95

**Published:** 2011-09-05

**Authors:** Bridget Kelly, Louise A Baur, Adrian E Bauman, Lesley King, Kathy Chapman, Ben J Smith

**Affiliations:** 1 Prevention Research Collaboration, School of Public Health, University of Sydney, Sydney, Australia; 2 Cancer Council NSW, Sydney, Australia; 3 Department of Health Social Science, Monash University, Victoria, Australia

**Keywords:** Food, Beverages, Child, Marketing, Sport, Sponsorship

## Abstract

**Background:**

Children's exposure to unhealthy food marketing influences their food knowledge, preferences and consumption. Sport sponsorship by food companies is widespread and industry investment in this marketing is increasing. This study aimed to assess children's awareness of sport sponsors and their brand-related attitudes and purchasing intentions in response to this marketing.

**Methods:**

Sports clubs known to have food sponsors and representing the most popular sports for Australian children across a range of demographic areas were recruited. Interview-based questionnaires were conducted at clubs with children aged 10-14 years (n = 103) to examine their recall of local sports club and elite sport sponsors, and their attitudes towards sponsors and sponsorship activities.

**Results:**

Most children (68%) could recall sponsors of their sports club, naming a median of two sponsors, including a median of one food company sponsor each. Almost half (47%) of children could recall any sponsors of their favourite elite sporting team. Children aged 10-11 years were more likely than older children to report that they thought about sponsors when buying something to eat or drink (P < 0.01); that they liked to return the favour to sponsors by buying their products (P < 0.01); and that sponsors were 'cool' (P = 0.02). Most children had received a voucher or certificate from a food or beverage company to reward sport performance (86% and 76%, respectively). Around one-third of children reported liking the company more after receiving these rewards.

**Conclusions:**

Children's high recall of food and beverage company sport sponsors and their positive attitudes towards these sponsors and their promotions is concerning as this is likely to be linked to children's food preferences and consumption. Limiting children's exposure to this marketing is an important initiative to improve children's nutrition.

## Background

There is an accumulating body of evidence about the nature and extent of food marketing and the negative effects of this marketing on children's food habits [1,2]. The most recent systematic review on the impact of food marketing to children, commissioned by the World Health Organization in 2008, found that food advertising has a modest impact on nutrition knowledge, food preferences and consumption patterns, with subsequent implications for weight gain and obesity [1].

Children are viewed by the food industry as a major market sector, having influence over their own purchases as well as that of their parents [3]. Developing brand loyalty at a young age also seeks to ensure lifelong product purchases [3]. From a psychological perspective, there is substantial evidence to suggest that children, especially those less than eight years of age, are highly vulnerable to marketing as they lack the necessary cognitive skills and experience to assess these messages critically [4]. Notably, this evidence is primarily based on children's understanding of television advertising and their ability to interpret marketing from other forms of media is relatively unknown.

Corporate sponsorship of events and organisations represents one form of food and beverage marketing to which children are exposed [5]. Sponsorship is one of the fastest growing forms of marketing; with industry expenditure on all sponsorship promotions increasing by 22% since 2007 to a global value of US$46.3 billion [6]. This growth in sponsorship expenditure exceeds that of other advertising and sales promotions [6].

As well, research quantifying the extent of sport sponsorship has identified that corporate sponsorship is widespread across all levels of sport and is predominantly for unhealthy products [7]. An analysis of 107 websites for elite and club level organisations in New Zealand identified 640 sponsors, with those promoting gambling, alcohol and food and beverages high in fat, sugar and/or salt being twice as prevalent as companies marketing products or services that were considered to promote good health [7].

Further, in a telephone survey of 108 community sports club officials in Australia by the authors, the majority of clubs (65%) reported receiving sponsorship funding [8]. Importantly of the 59 food and beverage company sponsors identified, the majority were considered to be less health promoting, based on criteria developed from a Delphi survey of experts in physical activity, nutrition, health promotion and sport delivery [8]. These criteria focused on the nutritional quality of the majority of products sold by the company and the exclusion of companies that sold alcohol. Further, this study found that the provision of vouchers and branded certificates to players was a frequently used promotional technique by food and beverage company sponsors [8].

Despite the high prevalence of food and beverage company sponsorship of children's and elite sports, there have been no published studies measuring the effect of this sponsorship on children and adolescents [9]. This study aimed to assess children's awareness and perceptions of sports club sponsors, and to gauge children's attitudes and behavioural intentions in response to this marketing. We hypothesised that children would have a high recall of sponsors of both their own sports clubs and of their favourite elite sporting teams, and that younger children would be more influenced by this sponsorship, in terms of the products they preferred, purchased and consumed.

## Methods

### Sampling

Sports clubs providing popular junior sports from three geographic areas, and known to have any food or beverage company sponsors, based on a previous survey by the authors [8], comprised the sample for this study. These sports clubs had been randomly sampled from a list of all eligible clubs in selected Local Government Areas within three large geographical areas in Australia: Sydney and Illawarra Statistical Divisions and the Canberra/Queanbeyan Statistical District. Details of this original sampling have been provided elsewhere [8]. Sports clubs represented some of the most popular organised sports for children aged 5 to 14 years in Australia, including outdoor soccer, netball, rugby league, outdoor cricket, basketball and athletics/track and field, according to Australian Bureau of Statistics data [10]. Children aged 10 to 14, who were members of the selected sports clubs and who had a consent form signed by themselves and/or a parent/guardian were approached to participate.

### Measures

A purpose-designed questionnaire was developed, based on previous surveys measuring children's awareness and recall of tobacco and alcohol company sport sponsorship [11-15], and attitudes towards sponsorship [16,17]. The questionnaire was initially piloted with a convenience sample of children (n = 5).

The questionnaire examined: i) the demographic characteristics of children (gender, age and suburb of residence); ii) unaided recall of and attitudes towards local sports club and elite sport sponsors; and iii) the value they place on sponsorship activities, including vouchers and branded certificates. Children were also asked to respond to a series of statements to determine their perceptions of sponsors; the perceived motivations of companies in sponsoring sport; and their beliefs about the effect of sponsorship on their purchasing and consumption behaviours. A Likert scale was used to indicate agreement with these statements: from 1 ('strongly agree') to 4 ('strongly disagree'). Recalled sponsors were cross-checked for accuracy with those sponsors reported by sports club officials and as listed on sporting organisations' websites.

The socio-economic status of children was determined according to the Australian Bureau of Statistics Socio-Economic Indexes for Areas (SEIFA) Index of Advantage\Disadvantage, using postcode of residence as a proxy measure [18]. SEIFA scores were stratified as high (> 1,100), medium (1,000-1,100) and low (< 1,000) socioeconomic areas.

### Procedures

Sports clubs were initially contacted by telephone and provided with written information to assess their interest in, and eligibility to participate in the survey. Clubs were then visited by a team of one to four interviewers between May and November 2010. Research officers were provided with a half-day training session, conducted by the lead investigator (BK), and all attended the first sports club visit together.

At each club approximately five children who were regular players were surveyed using convenience sampling, after they had returned a signed consent form. Ethics approval for this survey was granted by The University of Sydney Human Ethics Committee.

### Analyses

Data were entered into SPSS for Windows version 17.0 (SPSS Inc., Chicago IL.). Descriptive analyses including frequencies and cross-tabulations were used to describe recall of sponsors. Pearson's chi-square test was used to determine the significance of differences in responses to these variables by demographic group. The Mann-Whitney *U *test was used to assess differences in children's preferences and consumption of sponsoring companies' products, by age group and gender. Results were considered significant at the α = 0.05 level. Responses to open-ended questions were analysed thematically.

## Results

### Sample characteristics

The response rate for sports clubs was 95% (20/21), with only one soccer club declining participation. The overall sample was 103 children, with a mean age of 12 years (SD = 1.3). Most children (69%) played more than one organised sport, with children playing a median of two sports each (Interquartile range (IQR) = 1 to 3) (Table 1).

**Table 1:** Characteristics of children

	Children N (%)
Sport type attended	
Athletics	21 (20)
Basketball	5 (5)
Cricket	14 (14)
Netball	16 (16)
Rugby league	27 (26)
Soccer	20 (19)
Socioeconomic status of region	
Low (SEIFA < 1,000)	34 (33)
Medium (SEIFA 1,000-1,100)	50 (49)
High (SEIFA < 1,100)	19 (18)
Sex	
Female	42 (41)
Male	61 (59)
Age group	
10-11	45 (44)
12-14	58 (56)

### Awareness and recall of local sports club sponsors

Overall, 74% of children reported that they were aware of the companies and businesses that sponsored their sports club. A similar proportion of boys and girls reported that they were aware of club sponsors: 75% of boys vs. 69% of girls. Those children who were 10 years old were slightly less likely to be aware of club sponsors (67% vs. 75% for 11 to 14 year olds), although this difference was not significant (χ^2^_1 _= 0.7, P = 0.4).

In total, children recalled 119 current sports club sponsors, 22 regional sporting association sponsors and three past club sponsors. Of all correct current and past sport sponsors recalled, 51% were food and beverage companies, 39% were for non-food companies and 10% were alcohol-related businesses, including bars and clubs.

For those children who could correctly recall any sponsors, each child could recall a median of two sponsors (IQR = 1 to 3), including a median of one food sponsor (IQR = 0 to 2). These children could name 18% (IQR = 10 to 29) of all sponsors, and 33% (IQR = 10 to 29) of all food and beverage sponsors of their club. The majority of children who had reported that they could remember sponsors of their sports club were able to correctly name at least one sponsor (92%), and 68% could correctly name at least one food and beverage company sponsor.

### Awareness and recall of elite sport sponsors

Almost all children (n = 99) reported having a favourite elite professional-level sporting team. For these children, 59% reported that they were aware of the companies and businesses that sponsored this team. A total of 67 current team sponsors and two sponsors of sport development programs were recalled. Of the correct sponsors recalled, 84% were for non-food companies, 14% were food and beverage companies and 2% were alcohol-related businesses, including one alcohol manufacturer. As well, four companies for which the sports team had appeared in a television advertisement were mentioned.

A significantly greater proportion of boys reported that they were aware of the sponsors of their favourite sports team than girls, with 72% of boys reporting that they were aware compared to 40% of girls (χ^2^_1 _= 10.3, P < 0.001). There was no difference in awareness of sponsors between children of different ages.

For those children who correctly recalled any sponsors of their favourite elite sporting team (47% of all children), a median of one sponsor was recalled (IQR = 1 to 2), while most children (91%) did not recall any food and beverage sponsors. Most children (80%) who had said that they were aware of the team's sponsors could correctly name at least one sponsor, and 15% of these children named one or more food and beverage company sponsors.

### Perceptions of sport sponsors

Considering all local sports club sponsors named by children, including those that were correct and incorrect (n = 190 sponsors), the majority of children reported that they liked these companies 'a little' or 'a lot' (70%). A significantly greater proportion of children reported that they liked alcohol-related sponsors a lot compared to non-food or food and beverage companies (59% vs. 35% and 36%, respectively; χ^2^_6 _= 14.2, P = 0.03). Reasons given for liking sponsoring companies included enjoying the products sold by the companies (n = 42), and appreciating the companies' support of the club (n = 26), including through the provision of funding and equipment. Two children also liked these companies as they sponsored the premier league players for their sport.

Similarly, for both correctly and incorrectly recalled sponsors of elite sporting teams (n = 95), the majority of children liked these companies 'a little' or 'a lot' (70%). Reasons given for liking these companies included liking their products or services (n = 34), as well as the financial support of their team (n = 15).

### Perceptions of promotional activities

#### i. Vouchers

The majority of children (86%) had previously received a voucher from a food or beverage company to reward good sport performance. Of these children, 86% reported that they liked receiving the voucher either 'a lot' or 'a little'. There was no difference between children of different ages in their perceptions of these vouchers.

For those children who had received vouchers, 30% reported that they had liked the company more after they received this reward. A slightly greater proportion of younger children (aged 10 to 11) reported liking the company more after they received the voucher compared to older children (34% vs. 28% of 12 to 14 year olds; χ^2^_1 _= 0.5, P = 0.5).

#### ii. Certificates

Three-quarters of children (76%) had previously received a sporting certificate displaying a food or beverage company logo. The majority of these children also liked receiving these certificates (86%), while 38% liked the company more afterwards. A greater proportion of younger children reported that they liked the certificates a lot compared to older children (53% vs. 41%; χ^2^_2 _= 1.6, P = 0.4). Younger children also reported liking the company that provided this voucher more often than older children (41% vs. 36%), although this finding was not statistically significant (χ^2^_2 _= 1.6, P = 0.4).

### Food preferences and purchase intentions resulting from sport sponsorship

On a Likert scale ranging from 1 ('strongly agree') to 4 ('strongly disagree'), a median of 2 ('agree'), was reported for the statements:

- "I think food and drink companies that sponsor sport are cool";

- "I think that food and drink companies sponsor sport to help out sports clubs";

- "I like to return the favour to food and drink companies that sponsor my favourite sports by buying their products"; and

- "I think other children buy products because they sponsor their favourite sports".

Children also agreed that "food and drink companies only sponsored sport as a way of advertising" (median = 2).

Overall 85% of children thought that food and beverage companies sponsored sport to help out sports clubs ('strongly agree' or 'agree'), 69% thought that food and beverage sponsors of were 'cool', 66% thought that other children bought food and drink products because these companies sponsored their sport and 59% liked to return the favour to these sponsors by buying their products. Almost three-quarters of children (72%) thought that companies only sponsored sport to advertise their products.

**Figure 1:**
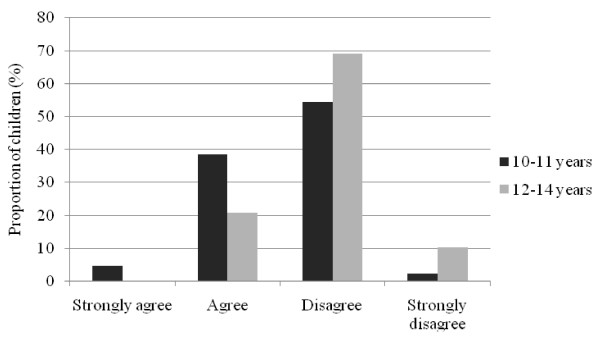
**Child responses to statement "When I'm in a shop, I think about if a food or drink company sponsors my favourite sports when I'm buying something to eat or drink", by age group**.

**Figure 2:**
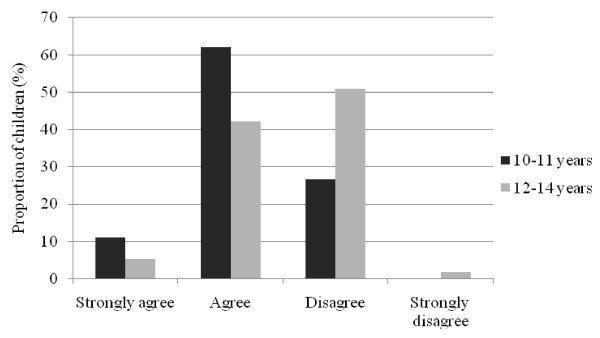
**Child responses to statement "I like to return the favour to food and drink companies that sponsor my favourite sports by buying their products", by age group**.

**Figure 3:**
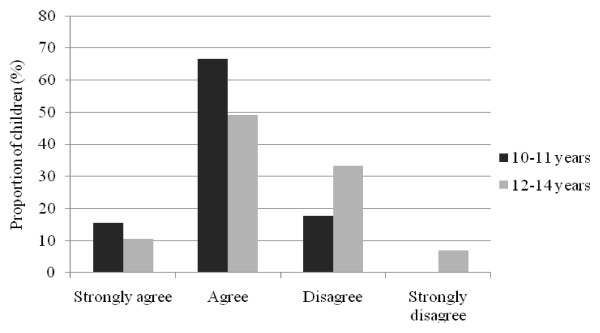
**Child responses to statement "I think food and drink companies that sponsor sport are cool", by age group**.

Younger children aged 10 to 11 years were significantly more likely to agree that they "thought about sponsors when buying something to eat or drink" compared to older children aged 12 to 14 years (median (Mann-Whitney *U *= 1627.0, *n*_1 _= 44, *n*_2 _= 58, P *<*0.01) (Figure 1). As well, younger children were more likely to agree that 'they liked to return the favour to sponsors by buying their products' (Mann-Whitney *U *= 1639.5, *n*_1 _= 45, *n*_2 _= 57, P < 0.01) (Figure 2); and thought that 'sponsors were cool' (median (Mann-Whitney *U *= 1596.0, *n*_1 _= 45, *n*_2 _= 57, P = 0.02) (Figure 3). There were no differences in responses by gender.

## Discussion

Findings from this survey indicate that children aged 10 to 14 years have a high awareness of corporate sponsors of their own sports clubs. More than two-thirds of children were able to recall at least one current or past sponsor of their sports club, and half were able to correctly name at least one current or past food and beverage company sponsor. Children were able to recall a greater proportion of all available food and beverage sponsors of sports clubs compared to non-food sponsors, suggesting that these food sponsors may have a greater resonance with children.

From our previous telephone survey with sports club officials relating to their sponsorship arrangements [8], sports clubs were more likely to sell or use food and beverage company sponsors' products at the club, offer sporting awards using these companies' logos and give vouchers for food and beverage company sponsors, compared to non-food companies [8]. Conceivably, these promotional opportunities are likely to be noticed and enjoyed by children. Indeed in the current survey, the majority of children had previously received vouchers and certificates from food and beverage companies and reported that they liked these rewards. Importantly, around one-third of children reported liking the food or beverage company more after receiving these rewards.

Fewer children were able to recall elite sport sponsors, with only around half of all children able to correctly name at least one sponsor of their favourite sporting team. For those children that were able to name an elite sport sponsor, the median number of sponsoring companies that were recalled was less than for children's own sports clubs. This is in contrast to findings from a survey of parents by the authors on the perceived effects of elite and local sport sponsorship on children [19]. In this survey, 86% of parents (n = 200) thought that elite sport sponsorship influenced the products that children liked, requested and purchased while less than half thought that sponsors of children's sports clubs influenced children. However, it is possible that children's heightened awareness of community sports club sponsors was due to their proximity to this setting at the time of the survey.

Children's awareness of sport sponsors, as indicated by their ability to recall sponsoring companies, is an indicator of their brand perceptions and potentially their consumption behaviours. Previous research relating to tobacco and alcohol sport sponsorship has indicated that children's awareness of brands is influenced by sponsorship, and that this awareness positively influences their perceptions and consumption of tobacco and alcohol products [20,21]. Research from the United Kingdom asked boys aged 12 and 13 years about their preferred sport, recall of cigarette brands and smoking behaviours in 1994 (n = 1,461) and again in 1995 (1,268) [20]. Boys who named car racing as their favourite sport were more likely than other boys to recall Marlboro (OR 1.91) and Camel (OR 1.92) cigarette brands, which were common sponsors for car racing. Those boys who named car racing as their favourite sport in 1994 were also significantly more likely to report that they smoked the following year (OR 1.96) [20].

Further, research from the United States has found that brand recognition and recall is a predictor of alcohol use. In one longitudinal study of 1,080 children, those with positive attitudes towards alcohol marketing and promotions had a 77% increased odds of drinking initiation compared to those who gave less positive assessments [21]. As well, higher brand recall was associated with a 10% higher drinking uptake at follow-up [21].

In the current survey, the majority of children reported that they liked both sports club and elite sport sponsors. In many cases, the reasons given for liking these companies were that they provided support to their club or favourite sports team. When asked about their responses to food and beverage company sport sponsorship, children mostly approved of these sponsors and thought that they were 'cool', as well as indicating that they bought sponsors' products to return the favour for supporting their sport. This was particularly the case for younger children. As well, while children recoginsed that sponsorship was an advertising activity for companies, they also mostly thought that companies were motivated by philanthropic intentions and wanted to assist sports clubs. Given the age range of children in this sample, these findings suggest that children's ability to interpret the commercial intent of sponsorship may occur at later ages compared to television advertising or may be hindered by other imputed motivations of sponsors.

Earlier research has found younger children to be more vulnerable to the effects of sponsorship [22,23]. Findings from qualitative research with children aged six to 16 years, in which children were presented with magazine advertisements showing tobacco company sponsorship of the Formula One Grand Prix, indicate that those under 10 years of age were less aware that the intention of the sponsorship was to promote cigarettes [22]. Similarly, in research from New Zealand that surveyed boys aged nine to 14 years (n = 302), those aged 13 and 14 were more likely to be aware that alcohol sponsorship of sporting events promoted alcohol, while younger children regarded this sponsorship as a charitable association [23]. However, in the current study there was no observed difference in younger or older children's perceptions of companies' motivations in sponsoring sport. As well, there were no significant differences in younger versus older children's perceptions of vouchers and certificates.

A major strength of this study was that interviews were conducted in the context of sport settings, thereby reaching those children who are actively engaged in community sports and most affected by sport sponsorship. While respondents were discouraged from looking around the club and at their uniforms during the survey, some children may have been able to visualise sponsors' logos at the club when asked to recall these. However, based on interviewer reports, this was not an issue in the majority of cases. Where it was evident that children had recalled a sponsor after viewing signage or a logo on their uniform, these sponsors were not recorded for that child.

A further limitation of this study was that questions relating to the effect of sponsorship on children's purchasing and consumption behaviours were based only on self-report. Objective evidence of the effect of sponsorship on actual product purchases is more equivocal and difficult to capture [24]. As well, the evaluation of sponsorship effects on product purchases is difficult to isolate from other marketing practices [12]. Nevertheless, such research is possible and future studies should seek to assess the effects of sponsorship on children's purchases using more empirical techniques, such as testing children's responses to sponsoring companies' products compared to those from non-sponsors. Research should also include children from a broader range of ages. As this study was based on children sampled from a small number of sports clubs and children were non-randomly sampled, there is also a need for further research to assess if the findings are consistent across a larger, more representative sample of children.

Finally, while the direct effects of sponsorship on children's product recall and product related attitudes and behavioural intentions is important, also of concern is the extent to which sponsorship by unhealthy companies creates an atmosphere of positive sentiments towards such products [23]. Research which captures how sponsorship can serve to enhance brand image and develop positive brand associations is also required [25].

Children's high level of recall of food and beverage company sport sponsors, and the positive attributes that children ascribe to these sponsors is concerning as this is likely to be linked to children's food preferences and consumption. This finding is particularly notable as many of these sponsors promote unhealthy products. Further, sponsorship activities, including vouchers and branded certificates, are attractive to children and can favourably influence children's brand perceptions. Limiting children's exposure to this marketing, either by restricting the types of companies that can sponsor sport or the types of promotional activities that can be used, would be an important obesity-prevention initiative to improve children's nutrition. Any policy intervention to limit this type of food marketing must also consider the viability of sporting clubs, such as through the provision of alternative funding mechanisms. Such an approach was successfully used in Australia with the advent of tobacco sponsorship restrictions [26], and could conceivably be applied here to limit children's exposure to unhealthy food and beverage company sport sponsorship.

## Competing interests

The authors declare that they have no competing interests.

## Authors' contributions

BK managed the data collection and analysis and drafted the manuscript. LAB, AEB, LK, KC and BJS provided strategic guidance for the study and the acquisition of funding. All authors were involved in the conception of the study, development of the surveys, and read and approved the final manuscript.
